# Chronic kidney disease and its health-related factors: a case-control study

**DOI:** 10.1186/s12882-021-02655-w

**Published:** 2022-01-10

**Authors:** Mousa Ghelichi-Ghojogh, Mohammad Fararouei, Mozhgan Seif, Maryam Pakfetrat

**Affiliations:** 1 Candidate in Epidemiology, Student Research Committee, Shiraz University of Medical Sciences, Shiraz, Iran; 2 HIV/AIDS research center, School of Health, Shiraz University of Medical Sciences, P.O.Box: 71645-111, Shiraz, Iran; 3 Department of Epidemiology, School of Health, Shiraz University of Medical Sciences, Shiraz, Iran; 4 Nephrologist, Shiraz Nephro-Urology Research Center, Department of Internal Medicine, Emergency Medicine Research Center, Shiraz University of Medical Sciences, Shiraz, Iran

**Keywords:** Chronic kidney disease, Related factors, Case-control

## Abstract

**Background:**

Chronic kidney disease (CKD) is a non-communicable disease that includes a range of different physiological disorders that are associated with abnormal renal function and progressive decline in glomerular filtration rate (GFR). This study aimed to investigate the associations of several behavioral and health-related factors with CKD in Iranian patients.

**Methods:**

A hospital-based case-control study was conducted on 700 participants (350 cases and 350 controls). Logistic regression was applied to measure the association between the selected factors and CKD.

**Results:**

The mean age of cases and controls were 59.6 ± 12.4 and 58.9 ± 12.2 respectively (*p* = 0.827). The results of multiple logistic regression suggested that many factors including low birth weight (OR _yes/no_ = 4.07, 95%CI: 1.76–9.37, *P* = 0.001), history of diabetes (OR _yes/no_ = 3.57, 95%CI: 2.36–5.40, *P* = 0.001), history of kidney diseases (OR _yes/no_ = 3.35, 95%CI: 2.21–5.00, *P* = 0.001) and history of chemotherapy (OR _yes/no_ = 2.18, 95%CI: 1.12–4.23, *P* = 0.02) are associated with the risk of CKD.

**Conclusions:**

The present study covered a large number of potential risk/ preventive factors altogether. The results highlighted the importance of collaborative monitoring of kidney function among patients with the above conditions.

## Background

Chronic kidney disease (CKD) is a non-communicable disease that includes a range of different physiological disorders that are associated with an abnormal renal function and progressive decline in glomerular filtration rate (GFR) [1–3]. Chronic kidney disease includes five stages of kidney damage, from mild kidney dysfunction to complete failure [4]. Generally, a person with stage 3 or 4 of CKD is considered as having moderate to severe kidney damage. Stage 3 is broken up into two levels of kidney damage: 3A) a level of GFR between 45 to 59 ml/min/1.73 m^2^, and 3B) a level of GFR between 30 and 44 ml/min/1.73 m^2^. In addition, GFR for stage 4 is 15–29 ml/min/1.73 m^2^ [4, 5]. It is reported that both the prevalence and burden of CKD are increasing worldwide, especially in developing countries [6]. The worldwide prevalence of CKD (all stages) is estimated to be between 8 to 16%, a figure that may indicate millions of deaths annually [7]. According to a meta-analysis, the prevalence of stage 3 to 5 CKD in South Africa, Senegal, and Congo is about 7.6%. In China, Taiwan, and Mongolia the rate of CKD is about 10.06% and in Japan, South Korea, and Oceania the rate is about 11.73%. In Europe the prevalence of CKD is about 11.86% [8], and finally, about 14.44% in the United States and Canada. The prevalence of CKD is estimated to be about 11.68% among the Iranian adult population and about 2.9% of Iranian women and 1.3% of Iranian men are expected to develop CKD annually [9]. Patients with stages 3 or 4 CKD are at much higher risk of progressing to either end-stage renal disease (ESRD) or death even prior to the development of ESRD [10, 11].

In general, a large number of risk factors including age, sex, family history of kidney disease, primary kidney disease, urinary tract infections, cardiovascular disease, diabetes mellitus, and nephrotoxins (non-steroidal anti-inflammatory drugs, antibiotics) are known as predisposing and initiating factors of CKD [12–14]. However, the existing studies are suffering from a small sample size of individuals with kidney disease, particularly those with ESRD [15].

Despite the fact that the prevalence of CKD in the world, including Iran, is increasing, the factors associated with CKD are explored very little. The present case-control study aimed to investigate the association of several behavioral and health-related factors with CKD in the Iranian population.

## Materials and methods

### Settings

In this study, participants were selected among individuals who were registered or were visiting Faghihi and Motahari hospitals (two largest referral centers in the South of Iran located in Shiraz (the capital of Fars province). Cases and controls were frequency-matched by sex and age. The GFR values were calculated using the CKD-EPI formula [16, 17].

### Data collection

An interview-administered questionnaire and the participant’s medical records were used to obtain the required data. The questionnaire and interview procedure were designed, evaluated, and revised by three experts via conducting a pilot study including 50 cases and 50 controls. The reliability of the questionnaire was measured using the test-retest method (Cronbach’s alpha was 0.75). The interview was conducted by a trained public health‌ nurse at the time of visiting the clinics.

Avoiding concurrent conditions that their association may interpreted as reverse causation; the questionnaire was designed to define factors preceding at least a year before experiencing CKD first symptoms. Accordingly participants reported their social and demographic characteristics (age, sex, marital status, educational level, place of residency), history of chronic diseases (diabetes, cardiovascular diseases, hypertension, kidney diseases, family history of kidney diseases, autoimmune diseases and thyroid diseases [18]). Also history of other conditions namely (smoking, urinary tract infection (UTI), surgery due to illness or accident, low birth weight, burns, kidney pain (flank pain), chemotherapy, taking drugs for weight loss or obesity, taking non-steroidal anti-inflammatory drugs, and taking antibiotic) before their current condition was started. Many researchers reported recalling birth weight to be reliable for research purposes [19]. Moreover, we asked the participants to report their birth weight as a categorical variable (< 2500 g or low, 2500- < 3500 g or normal, and > 3500 g or overweight). Medical records of the participants were used to confirm/complete the reported data. In the case of contradiction between the self-reported and recorded data, we used the recorded information for our study.

Verbal informed consent was obtained from patients because the majority of the participants were illiterate. The study protocol was reviewed and approved by the ethical committee of Shiraz University of Medical Sciences (approval number: 1399.865).

### Sample size

The sample size was calculated to detect an association‌ between the history of using antibiotics (one of our main study variables) and CKD as small as OR = 1.5 [20]. With an alpha value of 0.05 (2-sided) and a power of 80%, the required sample size was estimated as large as *n* = 312 participants for each group.

### Selection of cases

The selected clinics deliver medical care to patients from the southern part of the country. In this study, patients with CKD who were registered with the above centers from June to December 2020 were studied. A case was a patient with a GFR < 60 (ml/min/1.73 m^2^) at least twice in 3 months. According to the latest version of the International Classification of Diseases (2010), Codes N18.3 and N18.4 are assigned to patients who have (GFR = 30–59 (ml/min/1.73 m^2^) and GFR = 15–29 (ml/min/1.73 m^2^) respectively [21]. In total, 350 patients who were diagnosed with CKD by a nephrologist during the study period.

### Selection of the controls

We used hospital controls to avoid recall-bias. The control participants were selected from patients who were admitted to the general surgery (due to hernia, appendicitis, intestinal obstruction, hemorrhoids, and varicose veins), and orthopedic wards‌ from June to December 2020. Using the level of creatinine in the participants’ serum samples, GFR was calculated and the individuals with normal GFR (ml/min/1.73 m^2^) GFR > 60) and those who reported no history of CKD were included (*n* = 350).

### Inclusion criteria

Patients were included if they were ≥ 20 years old and had a definitive diagnosis of CKD by a nephrologist.

### Exclusion criteria

Participants were excluded if they were critically ill, had acute kidney injury, those undergone renal transplantation, and those with cognitive impairment.

#### Statistical analysis

The Chi-square test was used to measure the unadjusted associations between categorical variables and CKD. Multiple logistic regression was applied to measure the adjusted associations for the study variables and CKD. The backward variable selection strategy was used to include variables in the regression model. Odds ratios (ORs) and 95% confidence intervals (CIs) were calculated. All *p*-values were two-sided and the results were considered statistically significant at *p* < 0.05. All analyses were conducted using Stata version 14.0 (Stata Corporation, College Station, TX, USA).

## Results

In total, 350 cases and 350 age and sex-matched controls were included in the analysis. The mean age of cases and controls were 59.6 ± 12.4 and 58.9 ± 12.2 respectively (*p* = 0.83). Overall, 208 patients (59.4%) and 200 controls (57.1%) were male (*p* = 0.54). Also, 149 patients (42.6%) and 133 controls (38.0%) were illiterate or had elementary education (*p* = 0.001). Most cases (96.9%) and controls (95.7%) were married (*p* = 0.42). The mean GFR for CKD and control groups were 38.6 ± 11.4 and 78.3 ± 10.2 (ml/min/1.73 m2) respectively.

### Result of univariate analysis

Table 1 illustrates the unadjusted associations of demographic and health-related variables with CKD. Accordingly, significant (unadjusted) associations were found between the risk of CKD and several study variables including education, history of chronic diseases (diabetes, cardiovascular, hypertension, kidney diseases, autoimmune diseases, and hypothyroidism), family history of kidney diseases, smoking, UTI, surgery due to illness or accident, low birth weight, burns, kidney pain, chemotherapy, taking non-steroidal anti-inflammatory drugs, and taking antibiotics) (*P* < 0.05 for all).

**Table 1:** Unadjusted associations between demographic and health related variables with the risk of CKD

Variables	Cases (*N* = 350)	Controls (*N* = 350)	*P*-value*
	n (%)	n (%)	
**Sex**			
Female	142 (40.6)	150 (42.9)	0.54
Male	208 (59.4)	200 (57.1)	
**Age group**			
20- < 40	30 (8.6)	32 (9.1)	0.989
40- < 50	49 (14.0)	50 (14.3)	
50- < 60	89 (25.4)	90 (25.7)	
≥ 60	182 (52.0)	178 (50.9)	
**Place of residency**			
Rural	93 (26.6)	103 (29.4)	0.412
Urban	257 (73.4)	247 (70.6)	
**Education**			
Illiterate or elementary	149 (42.6)	133 (38.0)	0.001
Middle school	86 (24.6)	68 (19.4)	
High school	68 (19.4)	60 (17.1)	
College	47 (13.4)	89 (25.4)	
**Marriage status**			
Single	11 (3.1)	15 (4.3)	0.424
Married	399 (96.9)	335 (95.7)	
**Job**			
Employed	74 (21.1)	64 (18.3)	0.154
Unemployed	174 (49.7)	160 (45.7)	
Household	102 (29.1)	126 (36)	
**History of diabetes**			
No	181 (51.7)	292 (83.4)	0.001
Yes	169 (48.3)	58 (16.6)	
History of HTN**			
No	119 (34.0)	248 (70.9)	0.001
Yes	231 (66.0)	102 (29.1)	
**History of cardiovascular diseases**			
No	212(60.6)	290 (82.9)	< 0.001
Yes	138 (39.4)	60 (17.1)	
**History of kidney diseases**			
No	170 (48.6)	279 (79.7)	0.001
Yes	180 (51.4)	71 (20.3)	
**Family history of kidney diseases**			
No	250(71.43)	305 (87.1)	0.001
Yes	100(28.57)	45 (12.9)	
**History of smoking**			
No	207 (59.1)	233 (66.6)	0.003
Yes	143 (40.9)	117 (33.4)	
**Autoimmune diseases**			
No	319 (91.1)	335 (95.7)	0.015
Yes	31 (8.9)	15 (4.3)	
**Hypothyroidism**			
No	298 (85.1)	318 (90.9)	0.02
Yes	52 (14.9)	32 (9.1)	
History of UTI***			
No	232 (66.3)	288 (82.3)	< 0.001
Yes	118 (33.7)	62 (17.7)	
**History of surgery due to illness or accident**			
No	123 (35.1)	193 (55.1)	0.001
Yes	227 (64.9)	157 (44.9)	
**Low birth weight**			
No	316 (90.3)	338 (96.6)	0.001
Yes	34 (9.7)	12 (3.4)	
**History of burns**			
No	309 (88.3)	334 (95.4)	< 0.001
Yes	41 (11.7)	16 (4.6)	
**History of kidney pain**			
Not at all	192 (54.9)	231 (66.0)	< 0.001
Seldom	39 (11.1)	51 (14.6)	
Sometimes	62 (17.7)	45 (12.9)	
Mostly	57 (16.3)	23 (6.6)	
**History of chemotherapy**			
No	309 (88.5)	327 (93.4)	0.024
Yes	40 (11.5)	23 (6.6)	
**History of taking drugs for weight loss**			
No	326 (93.1)	336 (96.0)	0.095
Yes	24 (6.9)	14 (4.0)	
**History of taking non-steroidal anti-inflammatory drugs**			
No	203 (58.0)	254 (72.6)	0.001
Yes	147 (42.0)	96 (27.4)	
**History of taking antibiotic**			
Never	55 (15.7)	63 (18.0)	0.001
Rarely	154 (44.0)	222 (63.4)	
Sometime	100 (28.6)	57 (16.3)	
Frequently	41 (11.7)	8 (2.3)	

*Chi-square test; **HTN Hypertension; ***UTI Urinary tract infection

### Results of multivariable analysis

Table 2 illustrates the adjusted associations between the study variables and the risk of CKD. Most noticeably, low birth weight (OR _yes/no_ = 4.07, 95%CI: 1.76–9.37, *P* = 0.001), history of surgery (OR _yes/no_ = 1.74, 95%CI: 1.18–2.54, *P* = 0.004), family history of kidney diseases (OR _yes/no_ = 1.97, 95%CI: 1.20–3.23, *P* = 0.007), and history of chemotherapy (OR _yes/no_ = 2.18, 95%CI: 1.12–4.23, *P* = 0.02) were significantly associated with a higher risk of CKD. On the other hand, education (OR _college/illiterate or primary_ = 0.54, 95%CI: 0.31–0.92, *P* = 0.025) was found to be inversely associated with CKD.

**Table 2:** Adjusted association between the study variables and risk of CKD

Variables^a^	OR^b^	95% CI^c^	*P*-value
**Education**			
Illiterate or elementary	1	–	–
Middle school	1.41	0.85–2.31	0.174
High school	1.41	0.85–2.31	0.002
College	0.54	0.31–0.92	0.025
**History of diabetes**			
No	1	–	–
Yes	3.57	2.36–5.40	0.001
**History of hypertension**			
No	1	–	–
Yes	2.88	1.95–4.25	0.001
**History of kidney diseases**			
No	1	–	–
Yes	3.35	2.21–5.0	0.001
**Family history of kidney diseases**			
No	1	–	–
Yes	1.97	1.20–3.23	0.007
**History of surgery**			
No	1	–	–
Yes	1.74	1.18–2.54	0.004
**low birth weight**			
No	1	–	–
Yes	4.07	1.76–9.37	0.001
**History of chemotherapy**			
No	1	–	–
Yes	2.18	1.12–4.23	0.02
**History of taking non-steroidal anti-inflammatory drugs**			
No	1	–	–
Yes	1.66	1.12–2.47	0.012
**History of taking antibiotic**			
Never	1	–	–
Rarely	0.76	0.46–1.26	0.296
Sometime	2.03	1.13–3.36	0.018
Frequently	5.55	2.10–14.69	0.001

^a^The full model included education, history of chronic diseases (diabetes, cardiovascular, hypertension, kidney diseases, autoimmune diseases and hypothyroidism), family history of kidney diseases, smoking, UTI, surgery due to illness or accident, low birth weight, burns, kidney pain, chemotherapy, taking non-steroidal anti-inflammatory drugs, and taking antibiotics); ^b^*OR* odds ratio; ^c^*CI* confidence interval

## Discussion

The results of the present study suggested that several variables including, education, history of diabetes, history of hypertension, history of kidney diseases or a family history of kidney diseases, history of surgery due to illness or accident, low birth weight, history of chemotherapy, history of taking non-steroidal anti-inflammatory drugs, and history of taking antibiotics may affect the risk of CKD.

In our study, the level of education was inversely associated with the risk of CKD. This finding is in accordance with the results of a study conducted by K Lambert et.al, who suggested that illiteracy or elementary education may raise the risk of CKD [22]. The fact that education level is associated with health literacy, may partly explain our results that lower education and inadequate health literacy in individuals with CKD is associated with worse health outcomes including poorer control of biochemical parameters, higher risk of cardiovascular diseases (CVDs); a higher rate of hospitalization, and a higher rate of infections [23].

In the current study, the history of diabetes was associated with a higher risk of CKD. This finding is consistent with the results of other studies on the same subject [20, 21, 24–27]. It is not surprising that people with diabetes have an increased risk of CKD as diabetes is an important detrimental factor for kidney functioning as approximately, 40% of patients with diabetes develop CKD [27].

The other variable that was associated with an increased risk of CKD was a history of hypertension. Our result is consistent with the results of several other studies [20, 24, 25, 28]. It is reported that hypertension is both a cause and effect of CKD and accelerates the progression of the CKD to ESRD [29].

After controlling for other variables, a significant association was observed between family history of kidney diseases and risk of CKD. Published studies suggested the same pattern [24]. Inherited kidney diseases (IKDs) are considered as the foremost reasons for the initiation of CKD and are accounted for about 10–15% of kidney replacement therapies (KRT) in adults [30].

The importance of the history of surgery due to illness or accident in this study is rarely investigated by other researchers who reported the effect of surgery in patients with acute kidney injury (AKI), and major abdominal and cardiac surgeries [31, 32] on the risk of CKD. Also, AKI is associated with an increased risk of CKD with progression in various clinical settings [33–35]. In a study by Mizota et.al, although most AKI cases recovered completely within 7 days after major abdominal surgery, they were at higher risk of 1-year mortality and chronic kidney disease compared to those without AKI [31].

The present study also showed that low birth weight is a significant risk factor for CKD. This finding is consistent with the results of some other studies. However, the results of very few studies on the association between birth weight and risk of CKD are controversial as some suggested a significant association [19, 36, 37] whereas others suggested otherwise [36]. This may be explained by the relatively smaller size and volume of kidneys in LBW infants compared to infants that are normally grown [38]. This can lead to long-term complications in adolescence and adulthood including hypertension, decreased glomerular filtration, albuminuria, and cardiovascular diseases. Eventually, these long-term complications can also cause CKD [39].

Another important result of the current study is the association between chemotherapy for treating cancers and the risk of CKD. According to a study on chemotherapy for testicular cancer by Inai et al., 1 year after chemotherapy 23% of the patients showed CKD [40]. Another study suggested that the prevalence of stage 3 CKD among patients with cancer was 12, and < 1% of patients had stage 4 CKD [41, 42]. Other studies have shown an even higher prevalence of CKD among cancer patients. For instance, only 38.6% of patients with breast cancer, 38.9% of patients with lung cancer, 38.3% of patients with prostate cancer, 27.5% of patients with gynecologic cancer, and 27.2% of patients with colorectal cancer had a GFR ≥90 (ml/min/1.73 m^2^) at the time of therapy initiation [43, 44]. The overall prevalence of CKD ranges from 12 to 25% across many cancer patients [45–47]. These results clearly demonstrate that, when patients with cancer develop acute or chronic kidney disease, outcomes are inferior, and the promise of curative therapeutic regimens is lessened.

In our study, the history of taking nephrotoxic agents (antibiotics or NSAIDs drugs) was associated with a higher risk of CKD. Our result is following the results reported by other studies [48, 49]. Common agents that are associated with AKI include NSAIDs are different drugs including antibiotics, iodinated contrast media, and chemotherapeutic drugs [50].

### Strengths and limitations of our study

Our study used a reasonably large sample size. In addition, a considerably large number of study variables was included in the study. With a very high participation rate, trained nurses conducted the interviews with the case and control participants in the same setting. However, histories of exposures are prone to recall error (bias), a common issue in the case-control studies. It is to be mentioned that the method of selecting controls (hospital controls) should have reduced the risk of recall bias when reporting the required information. In addition, we used the participants’ medical records to complete/ confirm the reported data. Although the design of the present study was not able to confirm a causal association between the associated variables and CKD, the potential importance and modifiable nature of the associated factors makes the results potentially valuable and easily applicable in the prevention of CKD.

## Conclusions

Given that, chemotherapy is an important risk factor for CKD, we suggest the imperative for collaborative care between oncologists and nephrologists in the early diagnosis and treatment of kidney diseases in patients with cancer. Training clinicians and patients are important to reduce the risk of nephrotoxicity. Electronic medical records can simultaneously be used to monitor prescription practices, responsiveness to alerts and prompts, the incidence of CKD, and detecting barriers to the effective implementation of preventive measures [51]. Routine follow-up and management of diabetic patients is also important for the prevention of CKD. We suggest a tight collaboration between endocrinologists and nephrologists to take care of diabetic patients with kidney problems. In addition, surgeons in major operations should refer patients, especially patients with AKI, to a nephrologist for proper care related to their kidney function. Treatment of hypertension is among the most important interventions to slow down the progression of CKD [12]. Moreover, all patients with newly diagnosed hypertension should be screened for CKD. We suggest all patients with diabetes have their GFR and urine albumin-to-creatinine ratio (UACR) checked annually. Finally, the aging population and obesity cause the absolute numbers of people with diabetes and kidney diseases to raise significantly. This will require a more integrated approach between dialectologists/nephrologists and the primary care teams (55).

## Data Availability

The datasets generated and/or analyzed during the current study are not publicly available due to their being the intellectual property of Shiraz University of Medical Sciences but are available from the corresponding author on reasonable request.

## Abbreviations

CKD: Chronic kidney disease
ESRD: End-stage renal disease
GFR: Glomerular filtration rate
RRT: Renal replacement treatment
UTI: Urinary tract infection
OR: Odds ratios
CI: Confidence intervals
HTN: Hypertension
AKI: Acute kidney injury
